# Influence of pH and temperature on the performance and microbial community during the production of medium-chain carboxylic acids using winery effluents as substrate

**DOI:** 10.1007/s11356-024-33103-5

**Published:** 2024-04-01

**Authors:** Sharon B. Villegas-Rodríguez, Jorge Arreola-Vargas, Germán Buitrón

**Affiliations:** 1 Laboratory for Research On Advanced Processes for Water Treatment, Unidad Académica Juriquilla, Instituto de Ingeniería, Universidad Nacional Autónoma de México, Blvd. Juriquilla 3001, 76230 Queretaro, Mexico; 2 Department of Plant Pathology and Microbiology, Texas A&M University, College Station, TX 77843 USA

**Keywords:** Chain elongation, Caproate, Caprylate, Ruminal fluid, Winery effluents

## Abstract

Winery effluents containing high ethanol concentrations and diverse organic matter are ideal substrates for producing medium-chain carboxylic acids via fermentation and chain elongation. However, the process needs to be better understood. This study presents novel insights into the bioconversion mechanisms of medium-chain carboxylic acids by correlating fermentation and chain elongation kinetic profiles with the study of microbial communities at different pH (5 to 7) conditions and temperatures (30 to 40 °C). It was found that high productivities of MCCA were obtained using a native culture and winery effluents as a natural substrate. Minor pH variations significantly affected the metabolic pathway of the microorganisms for MCCA production. The maximal productivities of hexanoic (715 mg/L/d) and octanoic (350 mg/L/d) acids were found at pH 6 and 35 °C. Results evidence that the presence of *Clostridium*, *Bacteroides*, and Negativicutes promotes the high productions of MCCA. The formation of heptanoic acid was favor when *Mogibacterium* and *Burkholderia* were present.

**Graphical Abstract:**

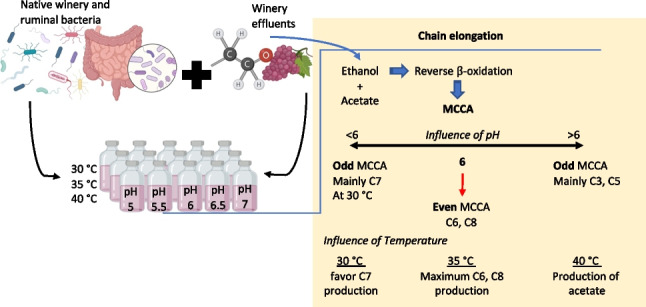

**Supplementary Information:**

The online version contains supplementary material available at 10.1007/s11356-024-33103-5.

## Introduction

In 2021, the International Organisation of Vine and Wine reported a wine production of 39.6 million liters in Mexico, ranking 37th as a wine producer worldwide (OIV 2021). That leads to a high generation of effluents since the total production of wastewater from a winery is about 1.2 times greater than the production of wine (Andreottola et al. 2009), generating substantial ecological risks if discharged without previous treatment. The effluents are characterized by a high content of complex organic molecules and high residual ethanol, up to 120 g/L (Vital-Jacome et al. 2020), resulting in a high organic matter concentration of 250 gCOD/L. Although the recovery of ethanol for valorization can be possible by distillation, the process leads to a high energy demand because of the high miscibility of ethanol in water (Xu et al. 2015).

On the other hand, these same qualities make wine effluents promising substrates to produce sustainable fuels and chemicals through the carboxylate platform, where ethanol can be used as an electron donor in the conversion of short-chain carboxylic acids (SCCA) such as acetate to medium-chain carboxylic acids (MCCA) containing between 6 and 12 carbon atoms, including a carboxylic group (De Groof et al. 2019). These acids are generally produced from fossil sources by chemical processes (Liu and Jarboe 2012).

In the biological process, SCCA are converted to MCCA through the reverse β-oxidation pathway, where certain types of bacteria use electron donors (ED) such as ethanol and lactate as carbon sources (Villegas-Rodríguez and Buitrón 2021). After production, the MCCA can be easily extracted from the fermentation broth due to its hydrophobic carbon chains. MCCA have diverse applications such as antimicrobials, corrosion inhibitors, bioplastics production, chemical industry precursors, biofuel (aviation fuels), or electrochemical hydrocarbons (Harvey and Meylemans 2014; Cavalcante et al. 2017; Urban et al. 2017; Reddy et al. 2018).

In terms of potential value, hexanoic acid has a market size of 25,000 tons per year, with an unrefined value of 1.18 USD/kg, five times higher than methane (0.24 USD/kg), and up to two times higher than ethanol (0.61 USD/kg), and refined value of $2000 to $3000 per ton (Kleerebezem et al. 2015; Zhang et al. 2020). Otherwise, the pH is one of the control factors in the chain elongation process. However, in open cultures, there needs to be more information about how the pH affects the microbial communities during MCCA production. The importance of pH relates to the cell stability damage caused by abrupt changes either by damage to the permeability of the plasma membrane and inhibition of enzymatic activity and transport systems or by alteration of the ionic state of the solutes to be transported (Roghair et al. 2018b).

Another factor currently limiting MCCA yields is competition with methane generation. The strategies to block methanogenesis without adding chemical inhibitors include pretreatment of the inoculum by heat shock to select spore-forming bacteria that are mainly fermentative (Steinbusch et al. 2009). However, a pretreatment could affect the presence of other microorganisms that, although they do not perform reverse beta-oxidation (RBO), maintain meaningful syntrophic relationships in the degradation of complex compounds or the formation of SCCA necessary to produce MCCA. That makes the pH one of the most critical factors in the chain elongation process. With the use of low pH values, it is possible to improve the efficiency of the process by minimizing the competition between reducing sulfate bacteria and methanogenic archaea, which are generally more sensitive to a lower pH (< 6) (De Groof et al. 2020). However, pH values under 5.5 favor the formation of MCCA in their undissociated form, which might bind with, displace, or block the connection between the electron carriers located within the cell membrane. Additionally, due to its hydrophobicity, a direct diffusion of the undissociated MCCA through the lipid membrane might occur (Cavalcante et al. 2017).

The maximum yield of hexanoic acid production by *Clostridium kluyveri* occurs at a pH of around 7 (Cavalcante et al. 2017). In addition, working at a pH close to neutrality favors the formation of MCCA in its dissociated form, which is not toxic to culture (Vasudevan et al. 2014). Therefore, a pH range between 5 and 6 could be suitable for producing hexanoic and octanoic acid because it inhibits most of the methanogenic activity, reducing the toxic effects of MCCA on the microbiota (Wang et al. 2014; Candry et al. 2020a).

Temperature control is another essential factor in any microbiological process, as it affects the growth rate of microorganisms. The elongation of the chain occurs at low temperatures (≤ 40 °C), and various authors suggest that the ideal pH and temperature conditions are not only related to the development of microorganisms and inhibition of competition for substrate but also vary according to the selected substrate type, where the solubility and hydrolysis of complex compounds present can be affected or favored according to these two factors (Kucek et al. 2016b; Xu et al. 2018). A recent winery waste study suggests that butyrate and MCCA are better produced using 37 °C (Hernández-Correa and Buitrón 2023). The objective of this study was to evaluate the effect of the pH and temperature on the production of medium-chain carboxylic acids, using winery effluents as substrate. A mixture of native microorganisms present in the winery effluents and ruminal microorganisms was utilized as inoculum.

## Methodology

The effect of pH and temperature in the fermentation of winery effluents for producing medium-chain carboxylic acids was investigated considering three temperature ranges: 30, 35, and 40 °C and five pH values from 5.0 to 7.0 (with a difference of 0.5 between each value) were evaluated for every temperature range.

### Inocula

The inoculum used was a combination of 80% native consortia present in the sediment of red winery effluents (Queretaro State, Mexico) and 20% ruminal fluid. The ruminal fluid was collected from a sheep slaughterhouse in Queretaro City. Both liquid and solid fractions were used, kept at 37 °C, and used within 2 h from sampling in the slaughterhouse. Total (TS) and volatile (VS) solids were determined in both inocula.

### Experimental conditions

Tests were conducted in serological bottles with a working volume of 80 mL. The winery effluents used as substrate (collected from a wine industry located in the state of Querétaro, México) came from the production of red wine. A physicochemical characterization of the effluent indicated a pH of 3.4; chemical oxygen demand (COD) total and soluble of 272.5 and 221.8 g/L; total and volatile solids of 74.6 and 49.1 g/L, respectively; total and volatile suspended solids of 52.1 and 9.7 g/L; and soluble carbohydrates of 2.0 g/L and 119.7 g ethanol/L. Before feeding to the serological bottles, the effluents were diluted with deionized water to achieve an ethanol-acetate ratio of 6/1 (23 g/5 g; 500/83 mmol or 90/10% COD. The medium was complemented with MES medium (4-Morpholineethanesulfonic acid monohydrate, CAS Number: 145224–94-8) to avoid variations in the established conditions. The pH was adjusted in each bottle with 5 M HCl. The bottles were inoculated with 1.6 g TS, giving a final concentration of 20 g TS/L, and N_2_ was sparged for 20 s to ensure anaerobic conditions. The bottles were placed in different incubators according to the corresponding temperature with orbital mixing at 150 rpm. The duration of all experiments (31 days) was defined by the concentration of ethanol available in the medium and by the concentrations of MCCA formed to avoid inhibition in the culture.

All measurements and tests were duplicated, and the results were expressed as mean ± standard deviation. ANOVA tests were conducted.

### Analytical methods

Liquid samples (2 mL) were collected every 48 and 72 h from the serological bottles to determine ethanol and carboxylic acids. The samples were centrifuged (600 g), and the supernatant was filtered using 0.45-µm nitrocellulose membrane filters. Samples were preserved by adding HCl to decrease the pH to 2 and then kept at 4 °C. A gas chromatograph (Agilent Technologies 7890B, USA) was used to analyze ethanol and carboxylic acids. It had a DB-FFAP column (15 m × 530 µm × 1 µm, Agilent) and an FID flame ionization detector. The initial temperature of the column was 60 °C; then, the temperature was gradually increased to 90 °C at a rate of 15 °C/min. Finally, the column temperature was increased to 170 °C at 25 °C/min. The initial temperature of the oven was 60 °C, the temperature of the injector was 190 °C, and that of the detector was 275 °C; nitrogen was used as the carrier gas. Total and volatile solids were measured according to standard methods (APHA 2005).

The volumetric rates to produce each carboxylic acid were evaluated by dividing the maximum concentration by the time needed to reach that concentration. The specific rate was calculated by dividing the volumetric rate by the total solids concentration of the inoculum added to each test.

### Microbial community analysis

At the beginning of the experiment (day 0), the inoculum was harvested to characterize the microbial composition. On day 31 of the experiment, only the biomass samples with the best MCCA production for each condition were microbiologically characterized once the fermentation was completed. The bottles were vigorously mixed for sampling, and 2-mL samples were collected in Eppendorf tubes. Then, the samples were centrifuged, and the pelleted biomass samples were stored at − 20 °C until further processing. Genomic DNA was extracted from biomass samples in a phosphate buffer. For DNA extraction, the PowerLyzer PowerSoil DNA isolation kit (QIAGEN, Hilden, Germany) was used according to the manufacturer’s instructions. DNA concentration was determined using NanoDrop 2000c equipment (Thermo Scientific, USA).

DNA samples were sent to the Integrated microbiome resource at Dalhousie University (Halifax, Nova Scotia, Canada) for amplification and sequencing through the MiSeq mass sequencing platform (Illumina, San Diego, USA). The universal primers used were B969F (ACGCGHNRAACCTTACC) and BA1406R (ACGGGCRGTGWGTRCAA), which amplified the V6-V8 regions of the 16 s rDNA targeting bacteria (Comeau et al. 2011; Willis et al. 2019; Vital-Jácome et al. 2023). The DNA samples’ integrity and concentration were evaluated using agarose gel stained with SYBR Green (1%) and quantified by spectrophotometry using a NANODrop 2000c (Thermo Scientific, USA).

Sequences were analyzed using Rstudio software (R Core Team 2021). Demultiplexed, error-free, and grouped sequences were obtained using the DADA2 algorithm (Callahan et al. 2016). The taxonomic composition of the samples was determined using the Silva-based 16S classifier (Quast et al. 2013). Lower rarefaction limits of 5379 sequences were applied to analyze bacterial sample diversity. The algorithm USEARCH was used to select only sequences no shorter than 100 pb and no longer than 250 pb with a quality score higher than 25 (Edgar 2013); chimeric sequences were eliminated using UCHIME (Edgar et al. 2011). The selected sequences were kept for community analysis. The taxonomic classification of each amplicon sequence variant (ASV) was performed using its consensus sequence, where the sequence was analyzed in the ribosomal database project (RDP) classifier by comparisons with high-quality sequences derived from the NCBI database (Wang et al. 2007).

## Results and discussion

### MCCA production

**Fig. 1:**
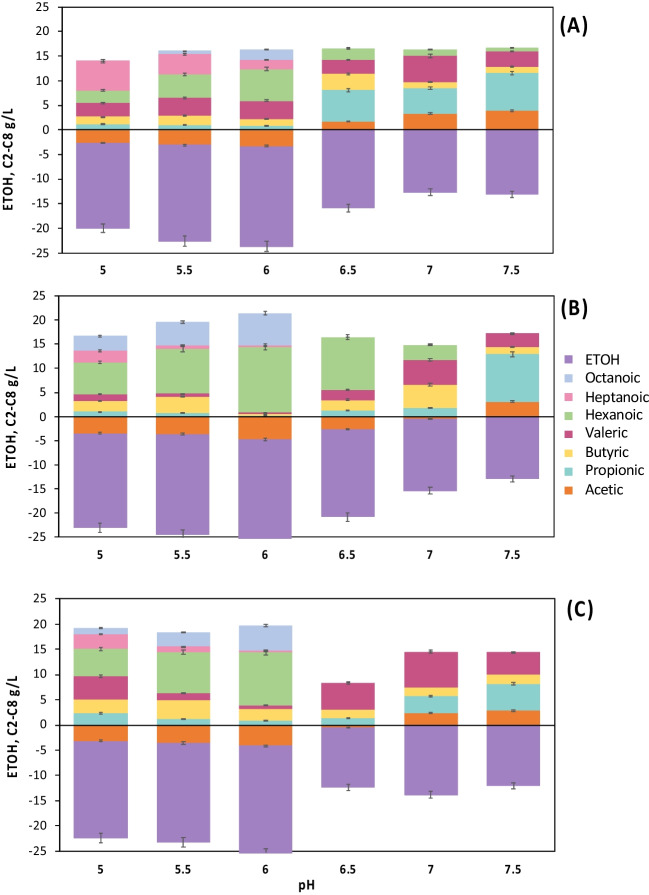
Production and consumption net during batch fermentation. Negative values indicate substrate consumption, while positive values indicate product formation. **A** 30 °C; **B** 35 °C; **C** 40 °C

The results of substrate consumption and production of net metabolites on day 31 are shown in Fig. 1 (See Table 1S in supplementary material). These are grouped according to the study temperature: group A (30 °C pH 5–7.5), group B (35 °C pH 5–7.5), and group C (40 °C pH 5–7.5). It was observed that both temperature and pH play an essential role in terms of the formation of MCCA and the conformation of the final metabolite formed, that is, even or odd.

The formation of odd acids (C3-C5) can be generated both in acidic and neutral conditions (group A, Fig. 1A); valeric acid (C5) is favored at pH 7 (5.25 g/L), while at pH greater than 7, the formation of (C3) is privileged (7.69 g/L). On the other hand, heptanoic acid (C7) production was only observed at acid pH values, and at pH 5, the highest acid concentration was obtained (5.97 g/L). Such values are higher than those reported in other studies. For instance, Grootscholten et al. (2013b) reported the production of heptanoic acid (3.2 g/L) when propionic acid (10.4 g/L) was added to a mixture of ethanol and acetate (ETOH /AA) ratio of 19.5/0.9 to promote elongation of valeric. Later, another research work reported the production of 1.5 g/L of heptanoic acid, but this time using the organic fraction of urban solid waste as substrate; as an electron donor, ethanol was applied (Grootscholten et al. 2013a). Roghair et al. (2018a) obtained a production of 1.8 g/L of heptanoic acid in a reactor fed with propionic acid (10.9 g/L/d) and ethanol (32.5 g/L/d). Zhang et al. (2020), separating the anaerobic digestion process into two stages, produced up to 2.7 g/L of C7 using pig manure as a substrate at pH 6.5.

Furthermore, it is essential to highlight that no external propionate was added for elongation. The residual presence of lactate (where rumen was used as inoculum) was the leading possible cause that could have favored the acrylate pathway under the conditions above. Although lactate leads to metabolic pathways like those of ethanol during the chain elongation process, the use of this electron donor has the disadvantage of the existence of a competitive acrylate pathway. Under that condition, lactate (when found in low concentrations) is converted to propionate, resulting in reduced selectivity for even MCCA production (Kucek et al. 2016a). For the formation of even MCCA as hexanoic acid (C6) and octanoic acid (C8), the pH range was more limited since, in all the cultures, the highest concentration was obtained at pH 6. Concentrations of 6.4 g/L for C6 and 2.01 g/L for C8 were reached at 30 °C.

In group B (Fig. 1B), the highest productions of C6 and C8 were obtained, reaching concentrations of up to 13.58 and 6.65 g/L, respectively. Similarly, it was observed that under this temperature condition (35 °C), pH closer to neutrality (pH > 6) favored the formation of propionic acid (1.24–9.81 g/L) and valeric acid (2.11–5.19 g/L). For C7 production, the highest production (2.45 g/L) occurred at pH 5; for this culture, the concentration was 2.5 times lower than the concentration obtained at 30 °C. At pH > 6, the formation of C7 or C8 was not carried out, and at pH 7.5, the C6 formation was completely inhibited. Candry et al. (2020b) found that variations in pH > 6 resulted in a propionic and acetic acid-producing community, while pH values < 6 generated a C6-producing community (3.7 g/L in 30 days). At pH 6.5, propionic acid was produced (8.84 g/L in 33 days). The hexanoic acid production obtained in this work was almost four times higher than the reported results, with the advantage of using a real substrate in a shorter period.

For the third temperature (group C, 40 °C, Fig. 1C), the highest concentration of C5 of all the cultures was obtained at pH 7, reaching a concentration of 7.02 g/L, which exceeded that obtained by others (Ganigué et al. 2019) who reported a maximum concentration of 5.3 g/L at 35 °C and pH 6.5. The highest concentration of C7 was obtained at pH 5, the same as the two previous groups (2.95 g/L), very similar to that obtained at 35 °C. The second-best productions of the entire experiment were obtained for hexanoic and octanoic acids. Unlike the two previous groups, the production of MCCA occurred only at an acidic pH of 6, where values up to 10.44 and 4.98 g/L for C6 and C8 were observed.

Figure 1 shows that more than 90% ethanol and acetic acid consumption was carried out, but only in the cultures at pH 6. As both substrates were not entirely consumed, it can be assumed that they were not the limiting reactant. An inverse relationship was observed between the pH increase and ethanol consumption. However, for acetate production, the pH increase was proportional to the increment of the produced amount, reaching concentrations of up to 3.89 g/L at pH 7.5 and 30 °C.

For group A, the highest consumption of ethanol (90.5%) and acetate (68.90%) was observed in the culture at pH 6. In the culture at pH 6 and 35 °C (group B), the highest consumption of both substrates was 97% for ETOH and 97.8% for acetate, and the lowest was at pH 7.5, which could justify the absence of MCCA under these conditions. Finally, in group C, there was a consumption of up to 95.13% for ETOH and 84.5% for acetate at pH 6. The lowest consumption of ETOH (52%) in this group was observed at pH 7.5. The acetate formation in all cultures at pH > 7 coincides with the results obtained by Luo et al. (2017), which showed that at pH values of 6.5–7, various species are producers of acetic and propionic acids from lactate.

Besides the influence of the different pH and temperature conditions, increasing MCCA production could cause inhibition. From day 19 until the end of the experiment, in some cultures, the C6 concentrations ranged from 4.7 to 5.4 g/L (cultures at 30 °C, pH 5 and 5.5). The same was true for the cultures at 40 °C (pH 5.5 and 6), where concentrations of C6 were up to 4 g/L of ethanol at day 19. It has been reported that hexanoic acid at high concentrations can result in toxicity to microbiota. An extensive range of hexanoic acid concentrations (0.8 to 8.6 g/L) in fermentation cultures has been reported in the literature (Steinbusch et al. 2011; Liu and Jarboe 2012; Jeon et al. 2013; Vasudevan et al. 2014; Weimer et al. 2015; Ganigué et al. 2016; Lonkar et al. 2016). Accordingly, separating hexanoic acid from fermentation media has been proposed to improve the final yield (Jeon et al. 2013).

### Metabolites production

**Fig. 2:**
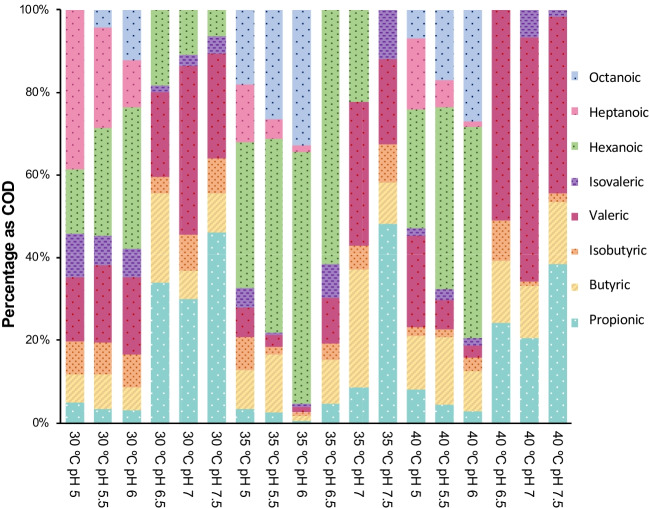
Percentage of the quantified metabolites (as COD) after 31 days for each condition studied

Figure 2 shows the COD percentage of the quantified metabolites after 31 days for each condition studied. It is crucial to remark that the pH plays a fundamental role in the formation of MCCA and in the conformation of the final metabolite formed (even or odd). As mentioned earlier, the pH range for producing even MCCA (C6-C8) was more limited, observing the highest concentration of C6 and C8 at pH values between 5.5 and 6.0 in all cultures. Regarding the temperature, it was observed that the formation of even MCCA is most favored at values > 30 °C. That could be justified because the optimal growth temperature for most C6-producing microorganisms, such as *C. kluyveri*, is reported at 35–37 °C. The highest percentage in COD of C6 and C8 (57.55 and 31.02%, respectively) was observed in the culture at 35 °C and pH 6.

In the formation of odd carboxylic acids, it is observed that the production can be carried out both in acidic and neutral conditions; however, only in acidic conditions is it possible to extend the chain to acids such as heptanoic acid. This metabolite obtained the highest percentage at 30 °C and pH 5 (27%). The production of acids of up to 5 carbons was only observed in the cultures at neutral pH. The culture at 40 °C and pH 7 showed the highest composition percentage of this acid (26.7%). But lower temperatures (< 35ºC) favor the concentration of these MCCA to a greater extent.

Ganigué et al. (2019) studied the production of valeric acid, separating the process into two stages; the first focused on propionic acid production using glycerol, and the second stage aimed at chain elongation by adding ethanol as an electron donor, a pure strain was used. Results showed that acetic and butyric acid favored the formation of even strings of MCCA, in low concentrations. Therefore, it is essential to avoid the presence of these two precursors if valeric acid production is wanted. That highlights the importance of the results obtained in this research to reveal how the pH could favor one or another route regardless of the intermediate metabolites present. The microorganisms in the inocula used in the current work show great sensitivity to minor pH variations.

**Fig. 3:**
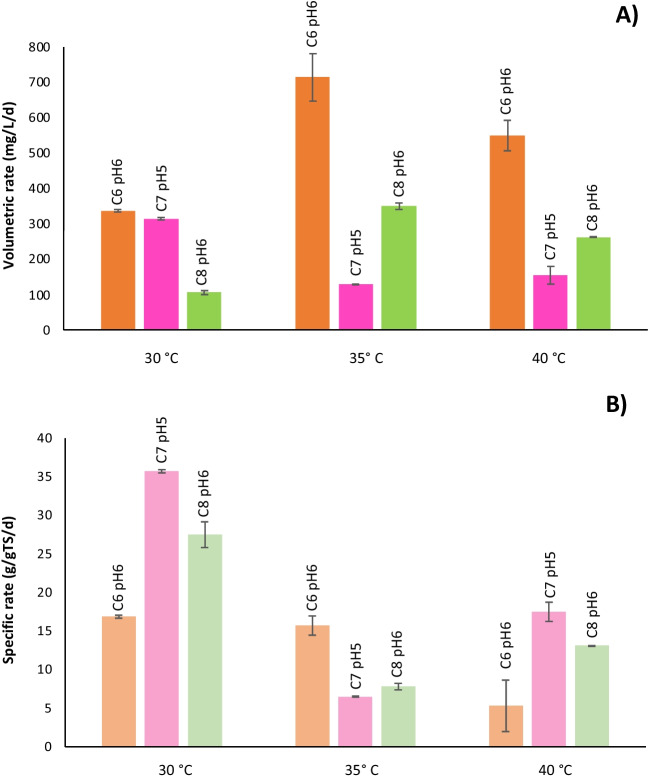
Average values for the maximum carboxylic acid: **A** volumetric and **B** specific formation rates in each temperature range

Figure 3 summarizes the pH values in which the maximum volumetric (Fig. 3A) and specific rates  (Fig. 3B) of MCCA were achieved in each temperature group studied. (Complete information for each carboxylic acid formed is shown in the supplementary information.) The best productivities of hexanoic acid (714.95 mg/L/d) and octanoic acid (349.97 mg/L/d) were obtained under pH 6 and a temperature of 35 °C. The highest hexanoic acid production rate observed was 5 to 7.23 times higher than in other studies working with solid organic waste (Grootscholten et al. 2013a; Bolaji and Dionisi 2017) and even up to 27.2 times higher than the value obtained in a continuous system using wine lees as substrate (Kucek et al. 2016c). At 35 °C and pH 6.5 and 40 °C and pH 6, concentrations of hexanoic acid were obtained (10. 85 and 10.44 g/L, respectively), like others (Duber et al. 2018) using acidic whey as a substrate.

The toxic limit of undissociated n-caproic acid for *Clostridium* sp. BS is 1.5 g/L (Andersen et al. 2017). Still, the concentration of that compound on day 6, when the formation of hexanoic acid was still observed, exceeded this value in all the cultures. Such results show that the microbiota was adapted to acidic environments with higher concentrations of undissociated hexanoic acid. That can be explained by microorganisms from winery effluents used as inoculum, which naturally adapted to low pH values and to these effluents’ inherent high ethanol content.

The highest productivity of heptanoic acid (314.11 mg/L/d) was obtained at 30 °C and pH 5. While for valeric acid, the highest productivity (369.47 mg/L/d) was obtained at 40 °C and pH 7. The response to the temperatures tested in this study indicated that the native microorganisms of winery effluents are more efficient at the temperature range of 35 to 40 °C for producing hexanoic and octanoic acid. Similarly, as previously reported, most reported caproic acid-producing microorganisms are mesophiles, such as *C. kluyveri* (Weimer and Stevenson 2012) and *M. elsdenii* (Marounek et al. 1989).

### Microbial community analysis

**Fig. 4:**
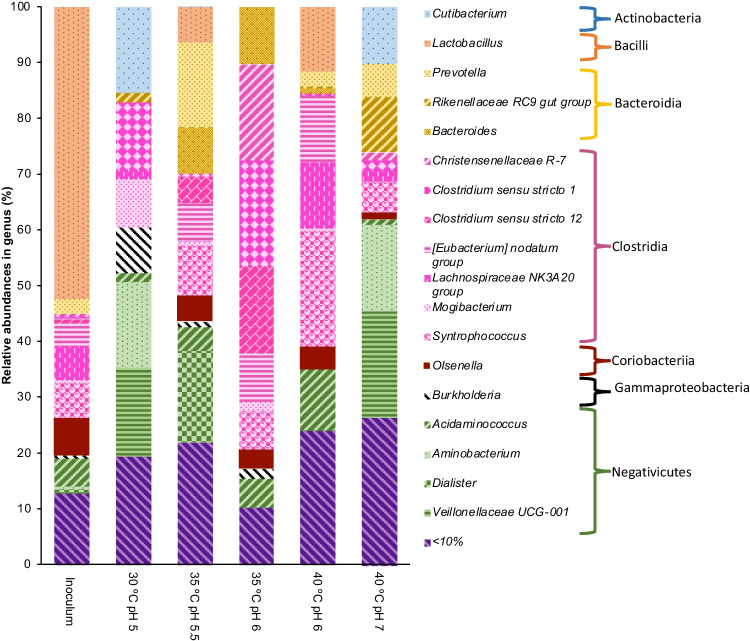
Relative abundances of the most abundant genus in all samples

For the microbial community analysis, five samples were selected according to the highest production of SCCA and MCCA (30 °C pH 5, 35 °C pH 5.5 and 6, 40 °C pH 6 and 7). In Fig. 4, the relative abundances by genus present in a percentage greater than 10% are shown for each of the analyzed cultures and grouped by color; the class to which they belong is indicated, where classes such as Clostridia (68.9%), Negativicutes (35.4%), and Bacteroidia (23.4%) presented the highest percentage of abundances in this experiment. Regarding the formation of microbial communities, a selection of microorganisms was observed in two main groups: the even MCCA producers and the odd SCCA and MCCA producers.

For the community that produces odd short-chain carboxylic acids, as mentioned above, it is observed that microorganisms present tolerance to both acidic and alkaline pH values. The culture at 30 °C and pH 5 showed significant similarity in its composition with the culture at 40 °C pH 7 with microorganisms belonging to the genera *Cutibacterium* (10–15%), which have been shown to produce acetic and propionic acid at values of pH of approximately 6.5–7 (Luo et al. 2017). Other genera, such as *Veillonella* (16–19%) and *Aminobacterium* (15–16%), were also found in both cultures (Candry et al. 2020b). Those authors found that pH values above 6 resulted in a community (dominated by these two genera) producing mainly propionic acid and acetic and valeric acid. However, the formation of heptanoic acid (C7) only took place at 30 °C pH 6 (5.97 g/L) when genera such as *Mogibacterium* (8.6%) and *Burkholderia* (8.2%) were present. That indicates that to produce C7, certain microorganisms are necessary and are only favored under acidic pH conditions (pH 5). Thus, although these cultures have not yet been reported as producers of MCCA, other microorganisms could be involved in lengthening. However, more specialized studies must be conducted to confirm this hypothesis.

In the even-numbered MCCA-producing community, the temperature and, to a greater extent, the pH turned out to be less flexible, with the highest production of C6 and C8 (13.58 and 6.65 g/L) obtained at 35 °C and pH 6 in a period of 21 days. Communities dominated by *Clostridium *sensu stricto* 1* (18.9%), *Clostridium *sensu stricto 12 (15.5%), *Christenellaceae-R7* (17.2%), and *Bacteroides* (10.3%) dominated the culture.

*Clostridium *sensu stricto* 1* and *Clostridium *sensu stricto* 12* communities were reported to be able to produce MCCA from ethanol (Candry et al. 2020a). These two genera were identified in all communities, and by using a consensus sequence in a BLAST search, they found a 100% match with *C. kluyveri DSM555* and *C. kluyveri 3231B.* Therefore, both can be attributed to the ability to produce hexanoic acid using ethanol from RBO. The presence of Clostridial in all cultures could indicate that this class is fundamental when ethanol is used as an electron donor, and the SCCA determines the direction of the pathway to even or odd MCCA presented in the medium. That can be selected by mainly the pH controlling the microorganisms forming these precursors. On the other hand, previous studies with similar substrates also revealed positive correlations between MCCA productivity and the relative abundance of OTUS for *Bacteroides* spp. when the electron donor was ethanol (Kucek et al. 2016c, a; Angenent et al. 2016).

According to the Spearman correlation (with a value of *p* < 0.05), a positive correlation was observed between the production of heptanoic acid and microorganisms of the genus *Burkholderia*, which could explain why only in the culture where this genus was detected was carried out the production of C7. Although the production of octanoic acid by *Clostridium *sensu stricto* 12* has not yet been reported. A positive correlation between this genus and the production of octanoic acid shows that this genus could produce octanoic acid in addition to hexanoic acid. Other positive correlations between the *Ruminococcus* and *Veillonela* genera with the production of valeric acid confirm the results reported by the authors mentioned above.

Observing the behavior of the different cultures under the various pH ranges provides another advantage of working with open cultures since, controlling this factor, the route towards the MCCA of interest can be selected, avoiding the 2-stage fermentation, which leads to more significant energy expenditure as well as the avoidance of specific strains in each stage. The origin of the inoculum could justify these results since they are microorganisms already adapted to the same winery effluents, which allowed them to ferment at high concentrations of ETOH without compromising the microbiota, having as an advantage that also makes it possible the use of exogenous ethanol as an electron donor.

## Conclusions

High productivities of MCCA were obtained using a native culture and winery effluents as a natural substrate. Minor pH variations significantly affected the metabolic pathway of the microorganisms for MCCA production implicating a great influence for the successful future industrial implementation of the process. The maximal productivity of hexanoic and octanoic acids was found at pH 6 and 35 °C. Results evidence the important presence of *Clostridium*, *Bacteroides*, and Negativicutes to promote high productions of MCCA. The formation of heptanoic acid was favor when *Mogibacterium* and *Burkholderia* were present.

## Supplementary Information

Below is the link to the electronic supplementary material.

Supplementary file1 (DOCX 45 KB)

## Data Availability

Data will be made available on a reasonable request to the corresponding author.
