# Supplementary material for: Influence of pH and temperature on the performance and microbial community during the production of medium-chain carboxylic acids using winery effluents as substrate
Source: Environ Sci Pollut Res Int. 2024 Apr 1;32(28):16617–26. doi: 10.1007/s11356-024-33103-5 (PMC12325424; doi:10.1007/s11356-024-33103-5)
Supplement: Supplementary file 1 — Supplementary file1 (DOCX 45 KB) [file 11356_2024_33103_MOESM1_ESM.docx]

**Influence of pH and temperature on the performance and microbial community during the production of medium-chain carboxylic acids using winery effluents as substrate**

Sharon B. Villegas-Rodríguez^a^, Jorge Arreola-Vargas^b^, Germán Buitrón^a*^

^a^Laboratory for Research on Advanced Processes for Water Treatment, Unidad Académica Juriquilla, Instituto de Ingeniería, Universidad Nacional Autónoma de México, Blvd. Juriquilla 3001, Queretaro76230, Mexico.

^b^Department of Plant Pathology and Microbiology, Texas A&M University, College Station, TX 77843, USA.

^*^Corresponding author:

Germán Buitrón

Laboratory for Research on Advanced Processes for Water Treatment, Unidad Académica Juriquilla, Instituto de Ingeniería, Universidad Nacional Autónoma de México, Blvd. Juriquilla 3001, Queretaro76230, Mexico.

Email: [gbuitronm@iingen.unam.mx](mailto:gbuitronm@iingen.unam.mx)

Tel. +52 (442) 1926165

**Supplementary Information**

**Table 1.** Average values and standard deviation (SD) for maximum carboxylic acid concentration and volumetric and specific formation rates for each tested inoculum.

|  | **Maximum concentration (g/L)** | | **Volumetric rate (mg/L/d)** | | **Specific rate (g/gTS/d)** | |
| --- | --- | --- | --- | --- | --- | --- |
|  | Average | SD | Average | SD | Average | SD |
| **30 ºC pH 5** | | | | | | |
| Butyric | 1.41 | 0.10 | 74.00 | 5.21 | 3.70 | 0.26 |
| Valeric | 2.80 | 0.01 | 147.37 | 0.37 | 7.37 | 0.02 |
| Isovaleric | 1.95 | 0.01 | 102.61 | 0.74 | 5.13 | 0.04 |
| Hexanoic | 2.58 | 0.01 | 135.58 | 0.74 | 6.78 | 0.04 |
| Heptanoic | 5.97 | 0.08 | 314.11 | 4.09 | 15.71 | 0.20 |
| Octanoic | 0.00 | 0.00 | 0.00 | 0.00 | 0.00 | 0.00 |
| **30 ºC pH 5.5** | | | | | | |
| Butyric | 1.87 | 0.01 | 98.45 | 0.74 | 4.92 | 0.04 |
| Valeric | 3.71 | 0.07 | 195.00 | 3.72 | 9.75 | 0.19 |
| Isovaleric | 1.41 | 0.02 | 74.21 | 1.12 | 3.71 | 0.06 |
| Hexanoic | 4.73 | 0.01 | 248.95 | 0.74 | 12.45 | 0.04 |
| Heptanoic | 4.11 | 0.02 | 216.08 | 1.12 | 10.80 | 0.06 |
| Octanoic | 0.73 | 0.04 | 38.16 | 2.23 | 1.91 | 0.11 |
| **30 ºC pH 6** | | | | | | |
| Butyric | 1.29 | 0.01 | 68.05 | 0.37 | 3.40 | 0.02 |
| Valeric | 3.80 | 0.04 | 200.00 | 2.23 | 10.00 | 0.11 |
| Isovaleric | 1.37 | 0.01 | 72.18 | 0.74 | 3.61 | 0.04 |
| Hexanoic | 6.40 | 0.07 | 337.05 | 3.72 | 16.85 | 0.19 |
| Heptanoic | 1.96 | 0.00 | 103.24 | 0.00 | 5.16 | 0.00 |
| Octanoic | 2.02 | 0.64 | 106.21 | 33.49 | 5.31 | 1.67 |
|  |  |  |  |  |  |  |
|  | **Maximum concentration (g/L)** | | **Volumetric rate (mg/L/d)** | | **Specific rate (g/gTS/d)** | |
|  | Average | SD | Average | SD | Average | SD |
| **30 ºC pH 6.5** | | | | | | |
| Butyric | 3.30 | 0.02 | 173.68 | 0.74 | 8.68 | 0.04 |
| Valeric | 2.84 | 0.07 | 149.47 | 3.72 | 7.47 | 0.21 |
| Isovaleric | 0.19 | 0.01 | 10.00 | 1.12 | 0.50 | 0.05 |
| Hexanoic | 2.33 | 0.03 | 122.63 | 0.74 | 6.13 | 0.07 |
| Heptanoic | 0.00 | 0.02 | 0.00 | 1.12 | 0.00 | 0.04 |
| Octanoic | 0.00 | 0.04 | 0.00 | 2.23 | 0.00 | 0.01 |
| **30 ºC pH 7** | | | | | | |
| Butyric | 1.23 | 0.04 | 64.74 | 0.74 | 3.24 | 0.01 |
| Valeric | 5.25 | 0.06 | 276.32 | 3.72 | 13.82 | 0.21 |
| Isovaleric | 0.35 | 0.01 | 18.42 | 1.12 | 0.92 | 0.08 |
| Hexanoic | 1.29 | 0.09 | 67.89 | 0.74 | 3.39 | 0.03 |
| Heptanoic | 0.00 | 0.07 | 0.00 | 1.12 | 0.00 | 0.05 |
| Octanoic | 0.00 | 0.01 | 0.00 | 2.23 | 0.00 | 0.09 |
| **30 ºC pH 7.5** | | | | | | |
| Butyric | 1.29 | 0.03 | 67.89 | 0.74 | 3.39 | 0.07 |
| Valeric | 3.10 | 0.08 | 163.16 | 3.72 | 8.16 | 0.05 |
| Isovaleric | 0.51 | 0.01 | 26.84 | 1.12 | 1.34 | 0.01 |
| Hexanoic | 0.73 | 0.03 | 38.47 | 0.74 | 1.92 | 0.02 |
| Heptanoic | 0.00 | 0.02 | 0.00 | 1.12 | 0.00 | 0.07 |
| Octanoic | 0.00 | 0.06 | 0.00 | 2.23 | 0.00 | 0.04 |
|  |  |  |  |  |  |  |
|  | **Maximum concentration (g/L)** | | **Volumetric rate (mg/L/d)** | | **Specific rate (g/gTS/d)** | |
|  | Average | SD | Average | SD | Average | SD |
| **35 ºC pH 5** | | | | | | |
| Butyric | 2.15 | 0.11 | 113.16 | 5.95 | 5.66 | 0.30 |
| Valeric | 1.44 | 0.18 | 75.82 | 9.30 | 3.79 | 0.47 |
| Isovaleric | 0.97 | 0.02 | 51.16 | 1.12 | 2.56 | 0.06 |
| Hexanoic | 6.62 | 0.01 | 348.16 | 0.74 | 17.41 | 0.04 |
| Heptanoic | 2.45 | 0.02 | 128.92 | 1.12 | 6.45 | 0.06 |
| Octanoic | 3.07 | 0.21 | 161.61 | 11.16 | 8.08 | 0.56 |
| **35 ºC pH 5.5** | | | | | | |
| Butyric | 3.31 | 0.07 | 174.21 | 3.72 | 8.71 | 0.19 |
| Valeric | 0.67 | 0.04 | 35.00 | 2.23 | 1.75 | 0.11 |
| Isovaleric | 0.12 | 0.03 | 6.50 | 1.49 | 0.33 | 0.07 |
| Hexanoic | 9.23 | 0.08 | 485.97 | 4.09 | 24.30 | 0.20 |
| Heptanoic | 0.88 | 0.01 | 46.05 | 0.37 | 2.30 | 0.02 |
| Octanoic | 4.74 | 0.04 | 249.45 | 1.86 | 12.47 | 0.09 |
| **35º C pH 6** | | | | | | |
| Butyric | 0.36 | 0.25 | 18.95 | 13.03 | 0.95 | 0.65 |
| Valeric | 0.30 | 0.03 | 15.61 | 1.49 | 0.78 | 0.07 |
| Isovaleric | 0.18 | 0.23 | 9.39 | 12.28 | 0.47 | 0.61 |
| Hexanoic | 13.58 | 1.27 | 714.95 | 66.99 | 35.75 | 1.25 |
| Heptanoic | 0.32 | 0.04 | 17.08 | 1.86 | 0.85 | 0.09 |
| Octanoic | 6.65 | 0.17 | 349.97 | 8.93 | 17.50 | 0.45 |
|  |  |  |  |  |  |  |
|  | **Maximum concentration (g/L)** | | **Volumetric rate (mg/L/d)** | | **Specific rate (g/gTS/d)** | |
|  | Average | SD | Average | SD | Average | SD |
| **35 ºC pH 6.5** | | | | | | |
| Butyric | 2.24 | 0.01 | 117.89 | 5.95 | 5.89 | 0.25 |
| Valeric | 2.11 | 0.19 | 111.05 | 9.30 | 5.55 | 0.54 |
| Isovaleric | 1.57 | 0.04 | 82.63 | 1.12 | 4.13 | 0.07 |
| Hexanoic | 10.85 | 0.03 | 571.05 | 0.74 | 28.55 | 0.03 |
| Heptanoic | 0.00 | 0.01 | 0.00 | 1.12 | 0.00 | 0.08 |
| Octanoic | 0.00 | 0.22 | 0.00 | 11.16 | 0.00 | 0.60 |
| **35 ºC pH 7** | | | | | | |
| Butyric | 4.80 | 0.08 | 252.63 | 3.72 | 12.63 | 0.16 |
| Valeric | 5.19 | 0.05 | 273.16 | 2.23 | 13.66 | 0.09 |
| Isovaleric | 0.00 | 0.02 | 0.00 | 1.49 | 0.00 | 0.05 |
| Hexanoic | 3.09 | 0.09 | 162.63 | 4.09 | 8.13 | 0.23 |
| Heptanoic | 0.00 | 0.03 | 0.00 | 0.37 | 0.00 | 0.30 |
| Octanoic | 0.00 | 0.08 | 0.00 | 1.86 | 0.00 | 0.08 |
| **35º C pH 7.5** | | | | | | |
| Butyric | 1.50 | 0.27 | 79.11 | 13.03 | 3.96 | 0.07 |
| Valeric | 2.78 | 0.02 | 146.32 | 1.49 | 7.32 | 0.06 |
| Isovaleric | 1.59 | 0.30 | 83.68 | 12.28 | 4.18 | 0.56 |
| Hexanoic | 0.00 | 1.10 | 0.00 | 66.99 | 0.00 | 2.30 |
| Heptanoic | 0.00 | 0.05 | 0.00 | 1.86 | 0.00 | 0.08 |
| Octanoic | 0.00 | 0.18 | 0.00 | 8.93 | 0.00 | 0.55 |
|  |  |  |  |  |  |  |
|  | **Maximum concentration (g/L)** | | **Volumetric rate (mg/L/d)** | | **Specific rate (g/gTS/d)** | |
|  | Average | SD | Average | SD | Average | SD |
| **40 ºC pH5** | | | | | | |
| Butyric | 2.86 | 0.16 | 150.53 | 8.19 | 7.53 | 0.41 |
| Valeric | 4.47 | 0.30 | 235.26 | 15.63 | 11.76 | 0.78 |
| Isovaleric | 0.30 | 0.19 | 15.79 | 10.05 | 0.79 | 0.50 |
| Hexanoic | 5.40 | 0.21 | 284.24 | 11.16 | 14.21 | 0.56 |
| Heptanoic | 2.95 | 0.47 | 155.29 | 24.93 | 7.76 | 1.25 |
| Octanoic | 1.18 | 0.49 | 62.08 | 26.05 | 3.10 | 1.30 |
| **40º C pH 5.5** | | | | | | |
| Butyric | 3.70 | 0.78 | 194.74 | 40.94 | 9.74 | 2.05 |
| Valeric | 1.40 | 0.06 | 73.68 | 2.98 | 3.68 | 0.15 |
| Isovaleric | 0.52 | 0.35 | 27.37 | 18.61 | 1.37 | 0.93 |
| Hexanoic | 8.12 | 0.28 | 427.16 | 14.89 | 21.36 | 0.74 |
| Heptanoic | 1.12 | 0.28 | 58.95 | 14.89 | 2.95 | 0.74 |
| Octanoic | 2.79 | 0.01 | 146.97 | 0.74 | 7.35 | 0.04 |
| **40 ºC pH6** | | | | | | |
| Butyric | 2.39 | 0.95 | 125.92 | 49.87 | 6.30 | 2.49 |
| Valeric | 0.67 | 0.30 | 35.00 | 15.63 | 1.75 | 0.78 |
| Isovaleric | 0.40 | 0.18 | 21.13 | 9.68 | 1.06 | 0.48 |
| Hexanoic | 10.44 | 1.27 | 549.37 | 43.15 | 27.47 | 3.35 |
| Heptanoic | 0.30 | 0.19 | 15.63 | 10.05 | 0.78 | 0.50 |
| Octanoic | 4.98 | 0.01 | 261.95 | 0.37 | 13.10 | 0.02 |
|  |  |  |  |  |  |  |
|  | **Maximum concentration (g/L)** | | **Volumetric rate (mg/L/d)** | | **Specific rate (g/gTS/d)** | |
|  | Average | SD | Average | SD | Average | SD |
| **40º C pH 6.5** | | | | | | |
| Butyric | 1.71 | 0.77 | 90.00 | 40.94 | 4.50 | 2.09 |
| Valeric | 5.23 | 0.05 | 275.32 | 2.98 | 13.77 | 0.17 |
| Isovaleric | 0.00 | 0.03 | 0.00 | 18.61 | 0.00 | 0.88 |
| Hexanoic | 0.00 | 0.19 | 0.00 | 14.89 | 0.00 | 0.79 |
| Heptanoic | 0.00 | 0.20 | 0.00 | 14.89 | 0.00 | 0.65 |
| Octanoic | 0.00 | 0.02 | 0.00 | 0.74 | 0.00 | 0.03 |
| **40º C pH 7** | | | | | | |
| Butyric | 1.71 | 0.71 | 90.00 | 40.94 | 4.50 | 1.98 |
| Valeric | 7.02 | 0.07 | 369.47 | 2.98 | 18.47 | 0.18 |
| Isovaleric | 0.81 | 0.31 | 42.63 | 18.61 | 2.13 | 0.87 |
| Hexanoic | 0.00 | 0.24 | 0.00 | 14.89 | 0.00 | 0.72 |
| Heptanoic | 0.00 | 0.30 | 0.00 | 14.89 | 0.00 | 0.80 |
| Octanoic | 0.00 | 0.04 | 0.00 | 0.74 | 0.00 | 0.06 |
| **40º C pH 7.5** | | | | | | |
| Butyric | 1.75 | 0.80 | 91.84 | 40.94 | 4.59 | 2.08 |
| Valeric | 4.39 | 0.08 | 231.05 | 2.98 | 11.55 | 0.12 |
| Isovaleric | 0.17 | 0.41 | 9.11 | 18.61 | 0.46 | 0.89 |
| Hexanoic | 0.00 | 0.30 | 0.00 | 14.89 | 0.00 | 0.69 |
| Heptanoic | 0.00 | 0.22 | 0.00 | 14.89 | 0.00 | 0.71 |
| Octanoic | 0.00 | 0.03 | 0.00 | 0.74 | 0.00 | 0.07 |
